# Opacité pulmonaire suspecte chez un patient atteint de sclérodermie systémique

**DOI:** 10.11604/pamj.2015.22.56.7663

**Published:** 2015-09-18

**Authors:** Youssef Kort, Naziha Khammassi

**Affiliations:** 1 Service de Médecine Interne, Hôpital Razi, Faculté de Médecine de Tunis, Tunis, Tunisie

**Keywords:** sclérodermie, anévrysme de l′artère pulmonaire, diagnostic, sclerodermia, pulmonary artery aneurysm, diagnosis

## Image en medicine

La sclérodermie systémique est une pathologie auto-immune non spécifique d'organe caractérisée par une sclérose cutanée qui peut être limitée aux doigts ou plus étendue. L'atteinte pulmonaire est le principal facteur de mauvais pronostic. L'hypertension artérielle pulmonaire primitive et l'atteinte interstitielle souvent à type de fibrose en sont les expressions les plus fréquentes. Patient de 53 ans chez qui le diagnostic de sclérodermie systémique a été porté devant des antécédents de nécrose digitale, un syndrome de Raynaud, une sclérodactylie, une hypertension artérielle pulmonaire (68mmHg) à l'échographie cardiaque et des anticorps antinucléaires positifs (fluorescence nucléolaire) à 1/80. Dans le cadre de la recherche d'une atteinte pulmonaire interstitielle et en dehors de toute symptomatologie respiratoire, une radiographie du thorax montrait une opacité médiastinale ronde de tonalité hydrique bien limitée en regard de l'arc moyen gauche. A ce stade, les diagnostics évoqués étaient un cancer broncho-pulmonaire (qui a une incidence élevée au cours de la sclérodermie), une adénopathie médiastinale et un anévrysme de l'aorte. Un complément d'angio-scanner thoracique objectivait une dilatation anévrysmale de l'artère pulmonaire gauche d'environ 47mm. L'abstention thérapeutique et la surveillance régulière étaient préconisées. Des anévrysmes de l'artère pulmonaire ou de ses branches ont été rapportés chez des patients atteints d'une hypertension artérielle pulmonaire. Le rôle d'une macroangiopathie n'est cependant pas exclu, puisque d'autres localisations anévrysmales aortiques et cérébrales ont été décrites chez des patients sclérodermiques. Ailleurs, les anévrysmes des artères pulmonaires peuvent être d'origine néoplasique, infectieuse, iatrogène (cathétérisme cardiaque) ou secondaire à une vascularite systémique (surtout la maladie de Behçet).

**Figure 1:**
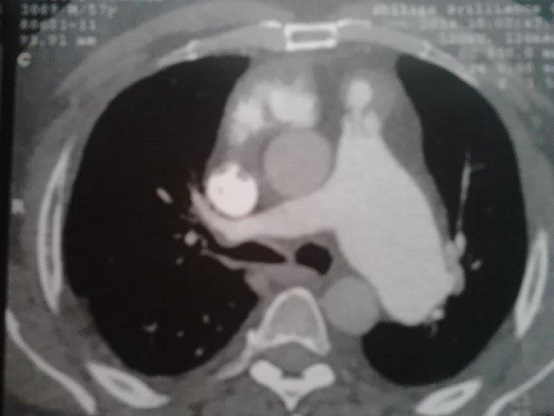
Angio-scanner thoracique: dilatation anévrysmale de l'artère pulmonaire gauche d'environ 47mm

